# Not bird-brained: Chickens use prior experience to solve novel timing problems

**DOI:** 10.1371/journal.pone.0282667

**Published:** 2023-04-05

**Authors:** Lisa Wiles, Sarah Cowie, Lewis Bizo

**Affiliations:** 1 Department of Psychology, University of Waikato, Hamilton, New Zealand; 2 School of Psychology, University of Auckland, Auckland, New Zealand; 3 Faculty of Business, Justice and Behavioural Sciences, Charles Sturt University, Bathurst, Australia; Tokai University, JAPAN

## Abstract

Despite differences between bird and human brain anatomy, birds have recently demonstrated capacities thought to be uniquely human, including planning and problem-solving. Many avian demonstrations of ‘complex’ behaviors rely on species-specific behavior (e.g., caching, tool use), or use birds who have evolved largely in similarly undomesticated circumstances (e.g., pigeons). In the present experiment, we asked how a species domesticated thousands of years ago, chickens (*Gallus gallus domesticus*), used past experience to navigate novel problems in the double-bisection task. The double-bisection task which has been used extensively with pigeons, allowing a comparison of signatures of chicken and pigeon performance on the same task. Our findings revealed chickens, like pigeons, show flexible learning that is sensitive to the broader context in which events occur. Further, as with pigeons, our chickens’ patterns of performance could be divided into two distinct categories which may reflect differences in the specific behaviors in which organisms engage during a timing task. Our findings demonstrate remarkable similarity in how chickens and pigeons use past experience to navigate novel problems. Further, these findings add to a growing body of knowledge suggesting the simplest forms of learning common across species–operant and respondent conditioning–are more flexible than is typically assumed.

## Introduction

In recent years, research has suggested animals possess capacities thought to be uniquely human. Perhaps particularly interesting is the observation of such capacities in birds, whose brain anatomy is distinctly different from that of mammals [1,2]. At least some birds are innovative tool manufacturers [3] and users [4] who can plan for the future [5–7] and engage in causal reasoning [8]. Others show remarkable long-term memory capacity [9–12], inference [10], episodic-like memory [11], and striking regularity in how they use past experience to navigate novel present conditions [13]. These avian displays of flexible, future-oriented behavior suggest that at least some elements of complex cognition are fundamental learning processes present in all species.

The ability to perceive the passage of time is critical in many complex behaviors [14]. For example, episodic memory is characterised by knowing one’s location in time, and planning for the future requires learning about extended temporal relations in one’s environment and adjusting behavior in accordance with what the present predicts about future events. Thus, the ability to learn about the relevance of time may be a species-general ability that is a fundamental building block of complex, flexible behavior. Understanding how animals learn the temporal structure of an environment, and the relation between time and other events, is therefore critical to understanding how flexible, future-oriented behavior develops. Models of time perception (e.g., [15–17]) are particularly useful in this endeavor; they make specific, testable predictions about how temporal learning occurs. Many of these models are based on ‘simple’ associative-learning mechanisms, but offer surprising flexibility in the range of behavior they can explain. One particularly promising model, Learning to Time (LeT [17]), asserts that animals learn to adjust their behavior in accordance with the passage of time when a behavioral state is repeatedly active at the point that a particular behavior produces a reinforcer, and other behavioral states are active at times when the same behavior does not produce a reinforcer. The more a state is associated with a reinforcer, the more it is likely to occasion the associated operant behavior. Associations between states and behaviors are strengthened by reinforcement *and* weakened by the absence of reinforcement, and hence temporal learning is context dependent and flexible.

**Fig 1:**
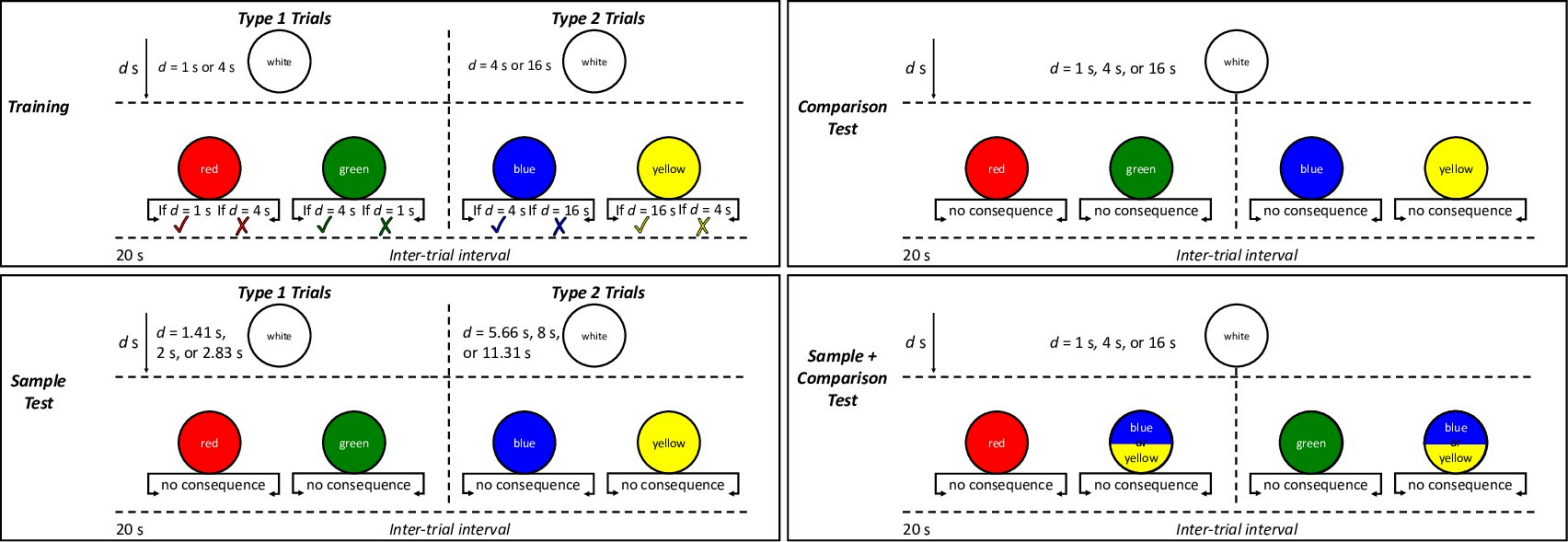
The double-bisection task. Note: Ticks denote correct choices, crosses incorrect. The durations and colors in the diagram are those used in the current study.

LeT provides a more accurate account of behavior than other models of timing in a number of lab-based timing procedures (e.g., [13]). One such procedure, the *temporal bisection task*, has been used extensively across different species including pigeons [13], rats, mice, and humans [18]. In the bisection task, animals learn during training to choose between two visually or spatially distinct *comparison stimuli* on the basis of which *sample duration* was presented immediately beforehand (Fig 1). Exposing animals to two types of trials during training (a *double-bisection task*) allows a particularly nuanced assessment of how animals learn about the relation between different durations and outcomes. In Type 1 training trials (Fig 1), choice of comparison stimulus A is correct following presentation of an *x*-s sample stimulus, and choice of comparison stimulus B is correct following presentation of a longer *y*-s sample stimulus. In Type 2 training trials, choice of comparison stimulus C is correct following presentation of a *y*-s sample stimulus, and choice of comparison stimulus D is correct following presentation of a longer *z*-s sample stimulus. Following training, some element of the task may be altered to assess how learning during training is applied to navigate situations that differ to some degree from those in the animal’s training history–for example, novel sample-stimulus durations (Sample Test trials; Fig 1), or novel combinations of comparison stimuli (Fig 1). Thus, the temporal-bisection task allows assessment of how learning acquired through extended training is used to solve familiar tasks and novel problems.

The double-bisection task is useful not only because it permits a test of different models of timing, but also because it lends itself to measures of behavior (‘signatures’) which are comparable across different species. The *psychometric function* plots the proportion of trials in which one of the two available comparison stimuli was chosen, as a function of the duration of the sample stimulus in that trial. Psychometric functions typically show a sigmoidal shift in preference for the stimulus associated with longer durations as the sample duration increases [13,19,20]. The steeper the psychometric function, the more pronounced the change in choice as the sample duration increases, and hence the stronger control by the sample duration. A logistic function [13,21] may be used to give a quantitative estimate of the slope and other characteristics of the psychometric function. A second measure, the *point of subjective equality* (PSE), reflects the sample duration that occasions equal choices of the two comparison stimuli. The closer the PSE falls to one of the two sample durations from training (i.e., the shorter or longer one), the less the learning from training with that sample duration generalizes to novel sample durations.

In the double-bisection task, signatures of performance are similar across different modalities including duration and length [22], and across species including pigeons, mice, and humans [18]. Extensive work with pigeons [13,19,23] has revealed two main patterns. First, functions from trials containing Type-1 and -2 stimuli do not superimpose, but there is individual variation in *how* the slopes of the functions differ. Most individuals show steeper functions in trials containing Type-2 stimuli (associated with longer times overall) than in trials containing Type-1 stimuli, but a minority show the reverse pattern or no difference. These features suggest pigeons learn the *relation* between the two sample-comparison pairs in each trial type, rather than sample-comparison pairs in isolation. Second, the PSE is typically slightly closer to the shorter sample than the longer one, falling at the geometric mean of the training durations for some subjects (as predicted by models of timing; e.g., [16]), and at even shorter durations for others [13,19]. Thus, learning from trials with longer samples tends to generalize more widely–perhaps because of increasing error in perception of longer durations [15].

Combining comparison stimuli in a novel way by including one stimulus from Type-1 training trials and another from Type-2 training trials (Comparison and Sample + Comparison Test trials; Fig 1) allows assessment of how past information from the sample duration is used in the face of an unexpected choice. In such situations, pigeons tend to choose whichever comparison stimulus has previously been ‘correct’ following a similar sample duration over a comparison stimulus never before associated with a similar sample duration, or one that has previously been incorrect following similar sample durations. When faced with a choice between a comparison stimulus that has never been associated with a similar sample duration, and one that has been explicitly incorrect follow a similar sample duration, pigeons tend to avoid the comparison they have learned as being explicitly incorrect. That is, the duration of the sample stimulus functions as a cue that signals both correct *and* incorrect behaviors, and hence learning from training can be applied flexibly to novel situations in which comparison stimuli do not contain an explicitly correct option [3,19,23–25]. Such patterns are generally consistent with LeT’s predictions about what is learned during a temporal discrimination task, and are certainly better explained by LeT than by other mainstream models of temporal learning (e.g., see [13], for discussion).

To provide a clearer picture of the ability of LeT to account for the processes that give rise to temporal learning across species, we examined the behavior of domestic chickens (*Gallus gallus domesticus*) following training on the temporal bisection task. While research has established (perhaps unsurprisingly) that chickens *can* discriminate the passage of time [26,27] it has not explored *how* they learn to do so. We therefore asked how chickens performed on the double-bisection task–a replication of Machado and Keen’s experiment [13] using a different species of subject. Understanding how chickens–often considered the ‘bird-brains’ of the avian world–learn in the double-bisection task provides a further test of LeT and its strengths and weaknesses. We assessed each individual chicken’s pattern of behavior under a range of novel situations, using a logistic function to provide a quantitative estimate of various aspects of behavior directly comparable to the behavior of pigeons on the same task [13,19,23–25]. A focus on the individual as its own control overcomes many of the constraints of psychological research that have led to replication failures [28,29], particularly those which create difficulties for comparative cognition (see [30] for discussion). Our findings add to the literature by demonstrating fundamental similarities in the way different avian species use past experience to solve familiar and novel tasks, and in the strengths and shortcomings of LeT in describing such learning across different species.

## Materials and methods

### Subjects

Three domestic Barnevelder hens numbered 10.1, 10.3 and 10.6 and three Crossbreed Bantam roosters numbered 10.2, 10.4 and 10.5 (all *Gallus gallus domesticus*) participated in the experiment. The hens all had the same prior experience pecking response keys for food on simple ratio schedules of reinforcement. The roosters had no prior experience pecking response keys for food. All birds were approximately two years of age at the start of the experiment. They were housed individually in wire cages that were approximately 500-mm long x 420-mm high x 500-mm wide in a ventilated room lit on a 12-hr light and 12-hr dark cycle. All birds were maintained at 80% ± 5% of their free-feeding body weight to ensure they were motivated to respond for the wheat reinforcers used in the experiment, maintained by post-session feeding of commercial pellets. All birds had free access to water in their cages, with grit and vitamin supplements provided weekly. The research was approved by the University of Waikato ethics committee (Protocol 894).

### Apparatus

An experimental chamber, which measured 615-mm long x 450-mm wide x 580-mm high, was used. The interior of the chamber was white with three keys and a food magazine mounted on the right-hand side of the chamber. The food magazine was located behind an opening (115-mm high x 70-mm wide) and centred 105-mm above the floor and when operated was lit by a 1-W light bulb.

Each response key was a frosted transparent Perspex key measuring 30-mm in diameter, positioned 390-mm from the floor and 85-mm apart in a horizontal position and could be lit by either a red, blue, yellow, green or white 28 –V multi-chip LED (light-emitting diode) bulb. Each effective key peck required a force of approximately 0.1 N and produced an audible beep that signalled key activation. When activated, a light above the magazine was illuminated, and the magazine was raised to allow access to wheat. All experimental events were controlled and recorded by a computer running MED-PC IV software.

### Procedure

We used a small-N design in which each individual hens experienced all conditions–that is, the individual functioned as its own control. A small-N design allowed us to assess the performance of individuals, and patterns across individuals, permitting a more direct comparison with previous research using different species. Given the observation of two distinct patterns of signature measures (see [13] for example), comparison at the group level would be inappropriate. Further, small-N designs are statistically powerful, and control for many of the factors that cause a failure to replicate [28–30].

Chickens first learned to choose one of two coloured stimuli according to the duration of a preceding sample stimulus, then were occasionally presented with longer or shorter sample stimuli, and/or with novel combinations of coloured stimuli. Our training and testing procedures followed those used by Machado and Guilhardi [13] as closely as possible in terms of the stimuli and structure of the training and experimental sessions (outlined in detail below). This was to ensure a fair comparison between chicken and pigeon behavior.

### Pretraining

Each pretraining session comprised 48 trials in which a sample duration was presented by lighting the center key white for some duration, and then the center key was turned off and two colored comparison stimuli were presented on the side keys. The correct comparison stimulus was always signalled by the duration of the sample stimulus (training; Fig 1). In Type 1 trials, the two-sample durations were 1-s and 4-s. Pecks to the red key following a 1-s sample, or to the green key following a 4-s sample, resulted in a reinforcer. In Type 2 trials, sample durations were 4-s and 16-s. Pecks to the blue key following a 4-s sample, or to the yellow key following a 16-s sample, produced a reinforcer. In both Type 1 and 2 trials, incorrect comparison choices resulted in the trial being repeated. Trials were separated by a 20-s inter-trial interval (ITI).

All birds were first trained on Type 1 trials until all could discriminate between both sample durations with 80% accuracy across repeated trials for ten consecutive sessions. Once this was achieved, all birds were trained in Type 2 trials with the same performance criteria as for Type 1 trials. Following mastery of both Type 1 and 2 trials, all birds received Type 1 and 2 trials across alternate sessions for a period of 8 to 38 days depending on individual accuracy. Finally, both Type 1 and 2 trials were presented in the same session. After all, birds had completed the training and achieved 80% accuracy across ten consecutive days (which took 10 to 20 sessions), the error-correction procedure was removed, so that incorrect color choices resulted in the beginning of the ITI. Following approximately ten sessions of this pretraining, chickens began training.

### Training

Training sessions comprised 48 trials that ended in a reinforcer for a correct response, and 24 extinction trials where the correct response ended the trial and initiated the ITI without access to a reinforcer. Type 1 and 2 trials (Fig 1; top panel) occurred in random order within a session. Extinction trials were introduced during training to ensure the absence of reinforcers in testing trials (which also ended without a reinforcer) would not be likely to signal a change in contingencies specific to the test-trial stimuli. Training continued for ten sessions before each type of Test began. In between each set of the Test sessions, the chickens were returned to training for five sessions. In the training sessions preceding the Stimulus-response-generalization tests, and the number of extinction trials increased from 24 to 32 because of the number of test trials required to display each different combination of stimuli in testing.

### Sample tests

In Sample test trials, novel sample stimuli of intermediate duration were introduced, and comparison stimulus color combinations were the same as in training (Sample Test; Fig 1). The sample-stimulus durations were logarithmically spaced: For Type 1 trials, sample stimuli were 1.41 s, 2 s, and 2.83 s long, and for Type 2 trials sample stimuli were 5.66 s, 8 s, and 11.31 s long. The middle duration of the test durations corresponded to the geometric mean of the training stimuli. Responses in test trials were never reinforced. Each test sample stimulus duration occurred four times in a session, and sample stimuli were presented on both left/right key color combinations. Thus, there were 24 test trials in each session.

### Comparison test

Comparison test trials used the same sample-stimulus durations as in training, but presented novel combinations of comparison-stimulus colors (Comparison Test; Fig 1). These new combinations were Red-Blue, Red-Yellow, Green-Bue, and Green-Yellow (i.e., one comparison stimulus from a Type 1 trial and another from a Type 2 trial). Each of these unique combinations of stimuli occurred twice per session. Each session comprised 56 regular trials and 24 test trials. Novel Comparison testing ran for 20 sessions.

### Sample + Comparison test

In Sample + Comparison tests, we presented novel sample-stimulus durations of either. 2- or 8-s, as well as the novel combinations of comparison stimuli used in Novel-Comparison trials (Sample + Comparison Test; Fig 1). The 8 test trials were presented four times within each session, twice for each left-key/right-key color combination, for 16 consecutive sessions. Due to an intermittent key-light problem caused by a loose wire, the Sample + Comparison test was repeated following an additional ten sessions of baseline.

## Results

### Sample tests

**Fig 2:**
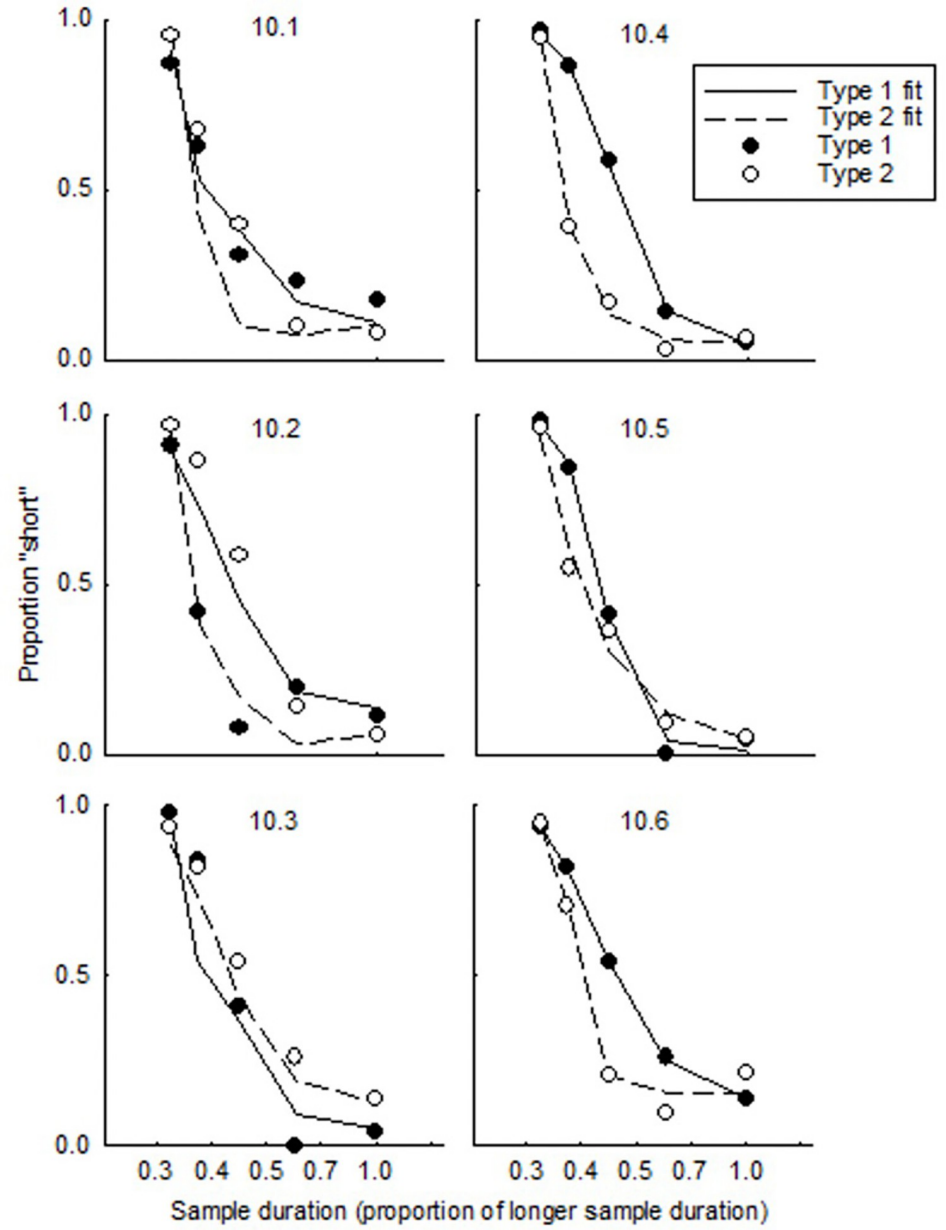
Probability of choosing short in sample tests. Note: Data are plotted as a function of the test sample duration relative to the longer sample-duration used in training. Circles show the proportion of responses made to the comparison stimulus associated with shorter durations during training; lines show the best fits of the logistic function (Eq 1) to the data.

Fig 2 shows the probability of choosing the comparison stimulus associated with a shorter sample duration (hereafter, the probability of *choosing short*) in Sample test trials, as a function of the duration of the test sample stimulus relative the longer training-sample duration. Filled data points denote data from trials with Type 1 comparison stimuli, and unfilled from Type 2. The solid lines are the best fits of a four-parameter logistic function that provides a quantitative description of how choice changed in response to variations in the sample-stimulus duration [3,21] (see [32] for a discussion about the utility of logistic functions for describing data):

$$P(short|t)={(y}_{0}-a)/\left[1+exp\frac{\left(T-\mu \right)}{\sigma }\right]$$ (1)

Eq 1 provided an excellent description of choice, accounting for between 98 and 100% of variance in the data. Table 1 shows the variance accounted for by Eq 1 for each individual, as well as the mean and standard deviation parameters from the fits.

**Table 1:** Parameters from fits of Eq 1 to individual data.

	Standard Deviation *σ*		Mean *μ*		Variance Accounted For	
Chicken	Type 1	Type 2	Type 1	Type 2	Type 1	Type 2
10.1	0.08	0.13	0.35	0.25	97%	100%
10.2	0.14	0.06	0.34	0.28	100%	99%
10.3	0.08	0.11	0.18	0.43	100%	97%
10.4	0.09	0.10	0.52	0.11	100%	100%
10.5	0.07	0.17	0.47	0.10	98%	100%
10.6	0.13	0.04	0.47	0.38	99%	100%

In Fig 2, the probability of choosing short decreased as the sample duration increased. Visual inspection of Fig 2 revealed a clear failure of superposition for all but Chicken 10.1. Fits of Eq 1 to data revealed smaller standard deviations (i.e., steeper functions) in Type 1 trials relative to Type 2 for four of six chickens; the other two showed the opposite pattern. A one-tailed paired-samples t-test did not reveal significant differences in the standard deviation t (5) = -.175, *p* = .868 or mean t (5) = 1.308, p = .248, and a Bayesian t test revealed anecdotal evidence for an absence of difference between the standard deviations (BF_10_ = .378) and between the means (BF_10_ = .689). The average of the mean from fits of the logistic function fell below the geometric mean of the training stimulus values for Type 1 (0.39, 95% CI [0.29,0.49]) and Type 2 trials (0.26, 95% CI [0.15,0.37]).

### Sample and Sample + Comparison tests

**Fig 3:**
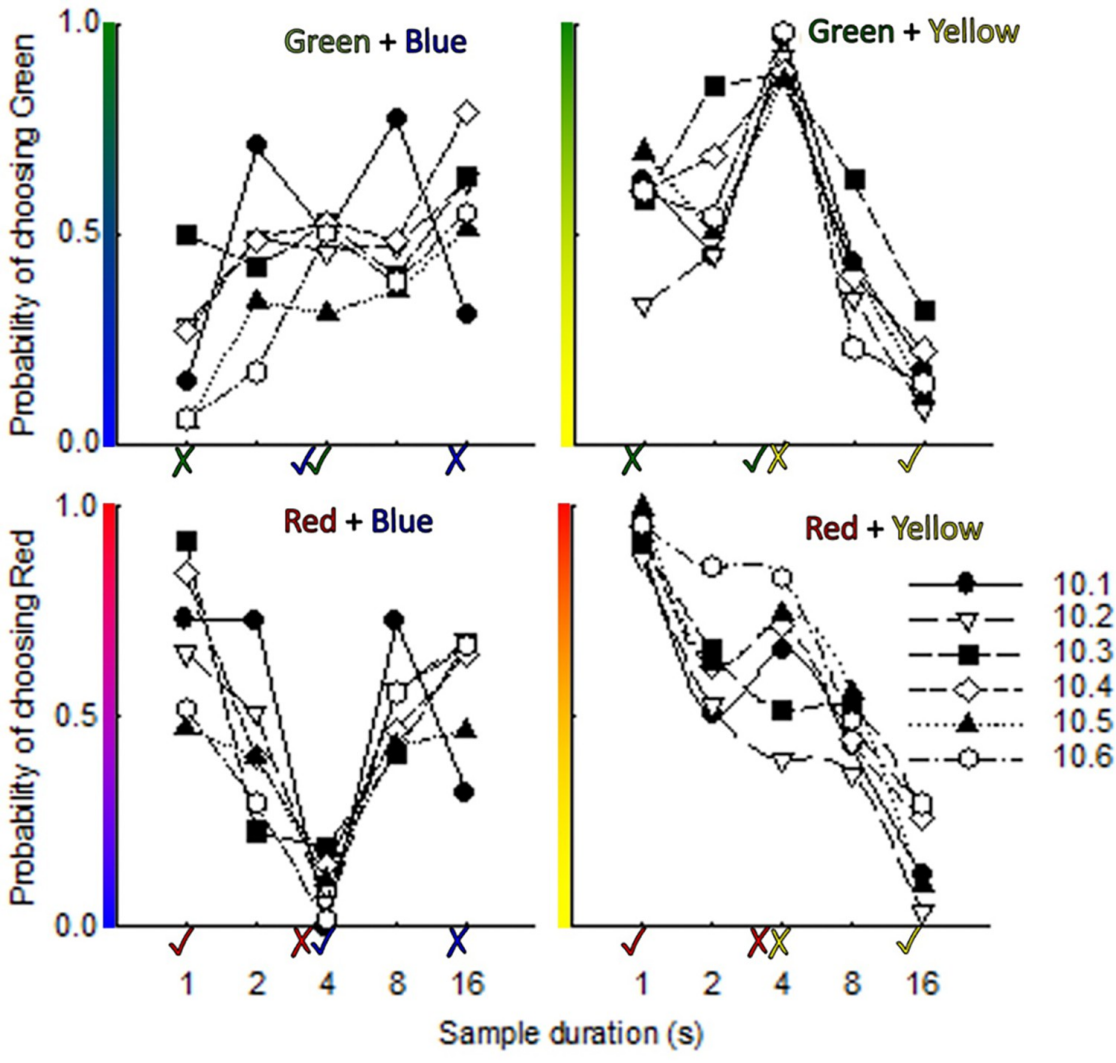
Probability of choosing comparison color in comparison and Sample + Comparison tests. Note: Colored ticks and crosses denote the sample duration at which choice of a comparison color was correct and incorrect (respectively) in training. Separate functions show data from individual chickens.

Fig 3 shows the proportion of responses to one of the two comparison stimuli in Sample and Sample + Comparison trials, for each novel combination of comparison stimuli as a function of sample-stimulus duration, for each chicken. Colored ticks and crosses below the x axis denote the sample duration at which a comparison color was correct and incorrect during training. From Fig 3, patterns of responding in a Test trial were strikingly similar across individual chickens.

### Green + Blue

In Green Blue test trials, choice for Green was most extreme following the longest sample duration, which during training had never been associated with Green but had signaled Blue was explicitly incorrect. Choice for Blue was most extreme following the shortest sample duration, which during training had never been associated with Blue but had signaled Green was incorrect. For all chickens except 10.1, choice was approximately indifferent (i.e., Blue and Green were chosen approximately equally) at the 4-s sample duration which had signaled Green and Blue were correct during training, and at the 2-s and 8-s durations which had never been associated with either color during Training.

### Red + Yellow

In Red Yellow trials, choice shifted from favoring Red to Yellow as the sample duration increased, with maximal choice for each color following the sample stimulus that had signaled the color as being correct during training (1 s for Red, 16 s for Yellow). For Chickens 10.2 to 10.4 and 10.6, choice shifted progressively from Red to Yellow over the intermediate sample durations which had either never been associated with either color (2 s, 8 s), or had signaled both Red and Yellow as being incorrect during training (4 s). For Chickens 10.1 and 10.5, choice following a 4-s sample stimulus favored Red to a greater extent than it had following the 2-s sample duration.

### Red + Blue

In Red Blue test trials, choice functions were v-shaped, with the strongest choice for Blue occurring following the 4-s duration associated with Blue being correct and Red being incorrect in training. Choice shifted progressively toward Red as the sample duration became more different from 4 s–either longer or shorter. For three chickens (10.1, 10.3, and 10.4), choice functions were asymmetrical, with choice for Blue being more extreme at the 1-s sample duration that had never been associated with Blue during training, relative to the 16-s sample duration that had explicitly signaled Blue was incorrect.

### Green + Yellow

In Green Yellow test trials, choice functions resembled an asymmetrical inverted v. The most extreme choice for Green occurred following a 4-s sample stimulus, which had signaled Green as being correct, and Yellow as incorrect, in training. The most extreme choice for Yellow following a 16-s sample duration, which had signaled Yellow as being correct, but was never associated with Green, in training. Following a 1-s sample stimulus, which had signaled Green was incorrect during training and had never been associated with Yellow, and a 2-s sample stimulus, which had never explicitly been associated with either color during training, choice was intermediate. Four chickens showed less extreme choice for Green following a 1-s sample than a 2-s sample; the other two showed the opposite pattern.

In general, then, Fig 3 shows stimuli that were in training associated with reinforcers after a similar or identical sample duration were more likely to be chosen over those never before associated with the same duration, and those that had explicitly been incorrect at a duration (i.e., were associated with no food). Generally, stimuli never before associated with a particular duration were more likely to be chosen than a stimulus that had been incorrect but associated with a duration during training.

## Discussion

We examined choices made by chickens on a double-bisection task following novel sample-stimulus durations (Sample Tests), and in the presence of novel combinations of comparison stimuli (Comparison Tests). This is the first study to assess chickens’ performance on the temporal-bisection task. Measures of chicken behavior in all types of Test showed patterns similar to those obtained from pigeons performing the same task [13,19,23–25]. Specifically, we found a failure of superposition of Type 1 and 2 psychometric functions, variation in the way functions failed to superimpose, and flexible application of past experience to solve novel problems in a manner consistent with LeT’s predictions. The similarity between chicken and pigeon behavior on the double-bisection task highlights generality in the way avian species with different evolutionary histories use past experience to navigate novel situations, and also underscores generality in both the strengths and weaknesses of LeT’s approach to explaining learning.

What does our chickens’ behavior tell us about how learning in a time-based task occurs? The patterns of behavior that we and others [3,19,23–25] have observed in the double-bisection task are generally consistent with predictions made by the Learning to Time (LeT; [17]) model of temporal learning. Just as pigeons’ psychometric functions on the double-bisection task fail to superimpose, so too did our chickens’ psychometric functions from Type 1 and 2 trials. A failure of superposition reflects differences in the accuracy of judgements about the sample duration in Type 2 and 1 trials, and hence violations of scalar timing. Similarly systematic violations of scalar timing have been reported in other studies [31–36]. LeT predicts a failure of superposition, but in a specific direction; because the overall reinforcer rate is constant, functions for Type 2 trials should be steeper than those in Type 1 trials.

Differences in the standard deviation (an estimate of slope) of each chicken’s Type-1 and -2 psychometric functions were consistent with LeT’s prediction for only two of our six chickens. For the remaining four, Type-1 psychometric functions were steeper than those in Type 2 trials. In pigeons [3,19,23–25], the direction of the difference is similarly variable across individuals. Our findings demonstrate that this variation is not a specific quirk of pigeon subjects, but is instead a more general outcome of temporal learning. The inability of LeT to account for a bi-directional difference in psychometric functions in the double-bisection task thus highlights a general shortcoming of the model (at least in its present form).

Temporal discrimination is typically more accurate and precise for shorter durations [15], although exceptions–including double-bisection-task performance–exist. These exceptions suggest temporal discrimination depends on more than the duration itself. Longer durations may create more opportunities to engage in temporally regular sequences of behavior which facilitate timing, but such sequences are not a requirement, and their occurrence and nature will thus depend on the individual. Certainly, temporal discrimination is improved in environments that facilitate engagement in other behaviors during the interval to be timed (termed *mediating* behaviors); performance on timing tasks worsens when animals are restrained [37], when space for movement is restricted [38], and when usual patterns of behavior are interrupted [39], and improves when mediating behaviors *must* be performed during the relevant duration [40], as well as when other behaviors are simply *able to* be performed [41]. Further, individuals who exhibit more behavior during an interval also perform more accurately [42], and obtain higher numbers of reinforcers [39]. When mediating behaviors are emitted at a different time from their usual occurrence, they tend to occasion incorrect timing responses [43]. So important are these mediating behaviors that humans report–albeit mistakenly–the entire *sequence* is necessary to produce reinforcers [44]. The impact of mediating behaviors on performance in time-based tasks suggests memory for one’s own behavior–episodic-like memory–is a critical component of learning the temporal structure of an environment, perhaps because such sequences act as an additional, enduring cue signalling the most appropriate behavior (see also [45]).

Although orderly, sequences of mediating behavior contain considerable variability [46], in terms of the time taken for each sequence to unfold, the evolution of orderly sequences with experience (e.g., [47]; see also topographical drift; [48]) and to some extent in the nature of the behaviors that make up each individual’s sequence. Such variation may cause imperfect temporal control [49], giving rise to systematic differences between the ability of different individuals to perform the same time-based task. Indeed, [13] noted that their pigeons with steeper Type-2-trial functions displayed a different pattern of behaviors before the comparison phase than did those with steeper Type-1 trials. It is reasonable to assume that had our chickens’ behavior been observed before the presentation of the comparison stimuli, we would have seen the same sort of differences in patterns of mediating behaviors according to whether a chicken’s performance different in tests with Type-1 and 2 stimuli. Given the apparent importance of mediating behavior in navigating the temporal structure of the world, it is essential to ask how these mediating behaviors develop, and why some individuals fill time with mediating behavior more efficiently than others. LeT’s conceptual approach to understanding the temporal organisation of behavior might capture the role of mediating behaviors in timing were such mediating behaviors able to be measured with the same rigor as are timing behaviors (e.g., see [50] for discussion).

As with psychometric functions from Sample Tests, LeT also makes predictions about functions in Sample + Comparison Tests are generally consistent with but not identical to actual individual psychometric functions. LeT asserts that learning in timing tasks is context-dependent because the associations between states and behaviors are both strengthened by reinforcement *and* weakened by the absence of reinforcement. We tended to observe a lack of systematic variation across intermediate sample-stimulus durations in Sample + Comparison tests (Fig 3) when LeT would predict a systematic variation (Green+Blue and Red+Yellow trials), and systematic variation when LeT would not predict it (Green+Yellow trials). [9] noted similar patterns in pigeons’ behavior in Red+Blue and Green+Yellow trials, although their pigeons’ response patterns in Green+Blue and Red+Yellow trials tended to conform more closely to LeT’s predictions than did our chickens’. Nevertheless, the similarity across species and studies in choice *not* predicted by LeT suggests that LeT may capture only some of the various processes underlying timing behavior.

One possible shortcoming of LeT is that it attributes all errors to temporal discrimination errors, even though discrimination of the relation the passage of time, behavior, and outcomes requires accurate detection of behaviors and outcomes, as well as the passage of time [51]. Indeed, the double-bisection task cannot be learned without detecting the relation between time and responses to stimuli of a particular color. Time is but one element of any environment; error in discriminating any non-temporal aspect of the environment would also cause weaker control. Such errors cannot be predicted or understood by a model of behavior whose sole focus is time perception. Where non-temporal discrimination errors occur, a model dealing only with temporal control will conflate non-temporal discrimination errors with temporal ones. This conflation will inadvertently creating timing errors [32,50,51]. The stream of environmental inputs an animal faces is distributed across both temporal and non-temporal dimensions. Navigating the world requires discrimination of what and where, as well as when–in this sense, even simple operant learning has episodic-like qualities that are not captured by mainstream theories (e.g., [52]) or models [17] of learning.

In conclusion, our findings show strong consistency in patterns of behavior in the face of novel situations across individual chickens. These patterns are similar to those observed in other studies with pigeons [13]–that is, findings appear similar across avian species with strikingly different evolutionary histories. These results add to a growing body of data demonstrating similarities in the way humans and non-human animals learn about the relation between the passage of time and other events [18]. Taken together, findings that animals use past experience flexibly to navigate new situations suggest that behavior does not merely fill time; behaviors take place at times when they are the best possible option either because past experience suggests the behavior is likely to produce a valuable outcome, or because it suggests other behaviors are *unlikely* to produce a valuable outcome. Organisms learn what events (stimuli, responses) will produce a particular consequence, and what will not. Such learning occurs in the context of time, and space, and other relevant dimensions, and as such as episodic-like in nature. Learning about time and how it relates to other events bears many similarities to learning about other dimensions (e.g., number, space; see for example [22]). Understanding how the organisation of behavior across time, space, and other relevant dimensions emerges, and why time and space are filled more efficiently in some environments and by some individuals, is key to understanding how simple learning underpins apparently complex behaviors.
